# Differential expression pattern of Bcl-2 family members in B and T cells in systemic lupus erythematosus and rheumatoid arthritis

**DOI:** 10.1186/s13075-023-03203-7

**Published:** 2023-11-22

**Authors:** K Kielbassa, L Van der Weele, AE Voskuyl, N de Vries, E Eldering, TW Kuijpers

**Affiliations:** 1 Department of Experimental Immunology, Amsterdam Infection & Immunity Institute (AIII), Amsterdam UMC, University of Amsterdam, Amsterdam, The Netherlands; 2 Department of Hematology, Cancer Center Amsterdam, Amsterdam UMC, University of Amsterdam, Amsterdam, The Netherlands; 3 Department of Lymphoma and Myeloma Center Amsterdam, Amsterdam, The Netherlands; 4 Department of Rheumatology and Clinical Immunology, Amsterdam Rheumatology and Immunology Centre (ARC), Amsterdam UMC, University of Amsterdam, Amsterdam, The Netherlands; 5 Department of Rheumatology and Clinical Immunology, Amsterdam Rheumatology and Immunology Centre (ARC), Amsterdam UMC, Vrije Universiteit, Amsterdam, The Netherlands; 6 Department of Pediatric Immunology, Rheumatology and Infectious Diseases, Emma Children’s Hospital, Amsterdam UMC, University of Amsterdam, Amsterdam, The Netherlands

**Keywords:** Systemic lupus erythematosus, Rheumatoid arthritis, Apoptosis, Bcl-2 family member expression

## Abstract

**Objective:**

This study aimed to evaluate the expression level of anti-apoptotic Bcl-2 family proteins in B and T cells in patients with systemic lupus erythematosus (SLE) and rheumatoid arthritis (RA) in relation to disease activity and the effect of various Bcl-2 family inhibitors (BH3 mimetics) as potential treatment.

**Methods:**

We included 14 SLE patients, 12 RA patients, and 13 healthy controls to study anti-apoptotic Bcl-2, Bcl-XL, and Mcl-1 expression and cell survival in different B and T cell subsets using stimulation assays and intracellular flow cytometry. Effect of various BH3 mimetics was assessed by cell viability analyses.

**Results:**

In SLE, significant differences in Bcl-2 family members were confined to the B cell compartment with decreased induction of Bcl-XL (*p* ≤ 0.05) and Mcl-1 (*p* ≤ 0.001) upon CpG stimulation. In RA, we did not observe any differences in expression levels of Bcl-2 family proteins. Expression patterns did not correlate with disease activity apart from decreased induction of Mcl-1 in B cells in active SLE. After in vitro stimulation with CpG, plasmablasts were more viable after treatment with three different BH3 mimetics compared to naïve or memory B cells in control and patient cells. After activation, Mcl-1 inhibition was most effective in reducing plasmablast and T cell viability, however, less in patients than controls.

**Conclusion:**

Our study provides evidence for the increased differential expression pattern of Bcl-2 family members in B and T cell subsets of patients with SLE compared to controls. Tested BH3 mimetics showed higher efficacy in controls compared to both autoimmune diseases, though nonsignificant due to low patient numbers.

**Supplementary Information:**

The online version contains supplementary material available at 10.1186/s13075-023-03203-7.

## Introduction

Apoptosis is critical for normal development and homeostasis of the immune system. There is emerging evidence that dysregulation of apoptosis is associated with autoimmune diseases [1, 2]. The commitment of lymphocytes to die is partly regulated by members of the Bcl-2 family [3]. Abnormal expression patterns of these proteins may promote not only apoptotic resistance of malignant cells [4–6] but also of pathogenic autoreactive lymphocytes leading to (progression of) autoimmune disease, as illustrated in several experimental autoimmune models [7–9]. A better understanding of the function and expression pattern of the major proteins involved is highly relevant since novel compounds that inhibit these proteins have entered the clinic. Currently, clinical application has been limited to hemato/oncology patients, yet these drugs may also be beneficial to patients with autoimmune disease. Since little is known about the expression pattern in even the most common (systemic) autoimmune diseases, we examined the role of three Bcl-2 family members, B cell lymphoma-2 (Bcl-2), B cell lymphoma extra-large (Bcl-XL), and myeloid cell leukemia-1 (Mcl-1) in systemic lupus erythematosus (SLE) and rheumatoid arthritis (RA).

SLE is a complex chronic inflammatory autoimmune disease characterized by widespread inflammation and tissue damage throughout multiple organ systems. The precise pathogenesis is still unknown, and the role of apoptosis appears to be complex. Bcl-2 transgenic mice provided the first experimental evidence that inhibition of apoptosis could lead to autoimmunity. Mice of the E mu-Bcl-2-22 transgenic strain had an increase in life span of B lymphocytes and plasma cells. Within the first year of life, most mice produced anti-nuclear antibodies (ANAs) and developed kidney disease, diagnosed as immune complex glomerulonephritis. Other clinical features included lymphadenopathy, elevated immunoglobulins, and myocardial infarction [7]. Another mouse study with a representative Bcl-2 transgenic strain revealed an increase in lifespan of anti-dsDNA B cells [8]. Both studies showed evidence of a lupus-like illness caused by continuous expression of Bcl-2, thereby inhibiting apoptosis of autoreactive B lymphocytes.

RA is a common autoimmune disease characterized by chronic inflammation and invasive growth of synovial tissue in the joints, resulting in cartilage and bone destruction. This leads to painful joint deformities and progressive disability. It is believed that impaired apoptosis of auto-reactive T and B cells in the peripheral blood could contribute to disease severity and progression [9]. Bcl-XL transgenic mice developed a progressive and more severe collagen induced arthritis (CIA) compared with wild-type control mice due to an enhanced Th1 response, which plays an arthritogenic role in both CIA and human RA [9–11].

Furthermore, there is evidence that Mcl-1 expression is essential for the long-term survival of plasma cells [12]. Auto-reactive plasma cells contribute to the production of auto-antibodies (e.g., rheumatoid factor (RF), anti-citrullinated protein antibodies (anti-CCP), and ANAs) which play a major pathogenic role in both RA and SLE. In RA and SLE, both T-cell-dependent and T-cell-independent pathways contribute to B-cell activation [13]. T-cell-dependent B-cell activation involves interactions between CD4+ T cells and B cells, resulting in the production of (auto)antibodies [14]. On the other hand, although T-cell-independent B-cell activation is generally considered less predominant, it occurs when B cells are directly stimulated by various factors, including Toll-like receptor (TLR) ligands like CpG DNA to lower the threshold of breaking or escaping normal mechanisms of B cell tolerance [15]. Moreover, these stimuli can robustly activate B cells, leading to the production of autoantibodies without requiring assistance from T cells. In the present study, we use B and T cell stimulation assays and intracellular flow cytometry to study Bcl-2, Bcl-XL, and Mcl-1 expression in different B and T cell subunits at basal levels and upon activation. We hypothesized that there is an increased expression of the Bcl-2 family members in peripheral blood B and T cells of patients with SLE and RA compared to healthy controls. This dysregulated expression promotes apoptotic resistance of pathogenic auto-reactive lymphocytes playing a role in the pathophysiology of the auto-immune disease. In addition, we examined in both diseases whether the expression pattern of the Bcl-2 family members correlates with disease activity. Furthermore, we studied the effect of several Bcl-2 family inhibitors (BH3 mimetics) that have been developed to treat in particular hemato-oncology patients as potential therapeutic targets for the treatment of both autoimmune diseases. In this study, we included the selective Bcl-2 inhibitor Venetoclax [16], the Mcl-1 inhibitor S63845 [17], and a novel dual Bcl-2 and Bcl-XL inhibitor AZD4320 [18].

## Materials and methods

### Ethics and informed consent

The study was approved by the local medical ethical committee of the Amsterdam University Medical Centers, location AMC (MEC_AMC_NL65076.018.15), and location VUmc (MEC_VUmc_ NL17200.029.07). All patients and healthy controls provided informed consent for study participation and publication of the related results. The study was performed according to the principles of the Declaration of Helsinki.

### Study subjects

For this cross-sectional study, we recruited 14 SLE patients, 12 RA patients, and 13 healthy controls who visited the Amsterdam UMC in Amsterdam, the Netherlands. All SLE patients fulfilled the American College of Rheumatology (ACR) 1997 classification criteria and all RA patients met the ACR 2010 classification criteria [17, 19]. Healthy controls were recruited at the Amsterdam UMC or obtained from Sanquin Blood Supply, Amsterdam, the Netherlands. Exclusion criteria covered patients with concomitant malignancies, other inflammatory diseases, or use of a biological or targeted synthetic disease-modifying anti-rheumatic drugs (DMARD) within the previous year (including anti-CD20 therapy). Sex, age, disease duration, medication use, disease activity, and clinical and laboratory parameters were annotated. As disease activity parameters, we used the SLE Disease Activity Index 2000 (SLEDAI-2k) [20] or Disease Activity Score 28-ESR (DAS28-ESR) [21]. Active SLE required a SLEDAI-2k score ≥ 6 with a clinical SLEDAI-2k (excluding laboratory results) score ≥ 4. Active RA required a DAS28-ESR score ≥ 2.6. Characteristics of the study participants are reported in Tables 1 and 2.

**Table 1:** Baseline characteristics of study participants

Baseline characteristics of the study participants			
	SLE patients (*n* = 14)	RA patients (*n* = 12)	Healthy donors (*n* = 13)
Demographic information			
Female, (%)	79	67	63
Age in years, median (IQR)	34.5 (21.8–47.5)	64.0 (48.8–69.0)	55.0 (35.8–66.8)

**Table 2:** Disease characteristics and laboratory data of SLE and RA patients

Disease characteristics and laboratory data of SLE and RA patients		
	SLE patients (*n* = 14)	RA patients (*n* = 12)
Years since diagnosis, median (IQR)	4.0 (0.8–7.8)	5.5 (1.5–9.3)
Medication		
None, *n* (%)	2 (14)	3 (25)
Prednisone, *n* (%)	1 (7)	1 (8)
Prednisone + cDMARD, *n* (%)	3 (21)	1 (8)
Prednisone dose, mg/day, mean (SD)	12.5 (2.0)	7.5 (3.5)
Antimalarials, *n* (%)	10 (71)	0 (0)
Azathioprine, *n* (%)	3 (21)	0 (0)
Methotrexate, *n* (%)	0 (0)	8 (67)
Mycophenolate mofetil, *n* (%)	2 (14)	0 (0)
Disease activity parameters		
DAS28-ESR, median (IQR)		2.5 (2.0–3.6)
SLEDAI-2k, median (IQR)	2.0 (0.0–9.3)	
Clinical parameters		
Extra-articular manifestations, *n* (%)		1 (8.3%)
SLEDAI-2k, organ system involvement, *n* (%)		
CNS	0	
Vascular	0	
Musculoskeletal	2 (14)	
Renal	2 (14)	
Mucocutaneous	3 (21)	
Cardiovascular and respiratory	2 (14)	
Immunological	10 (71)	
Constitutional	0	
Hematological	1 (7)	
Laboratory parameters		
RF positive, *n* (%)	5 (39)	10 (83)
Anti-CCP positive, *n* (%)	0	12 (100)
ANA positive, *n* (%)	14 (100)	1 (11)

Missing data SLE patients: RF (*n* = 1), anti-CCP (*n* = 2). Missing data RA patients: ANA (*n* = 3)

*ANA* Antinuclear antibody, *anti-CCP* Anti-cyclic citrullinated peptide, *CNS* Central nervous system, *DAS28-ESR* Disease Activity Score 28-erythrocyte sedimentation rate, *cDMARDS* conventional disease-modifying antirheumatic drugs, *RA* Rheumatoid arthritis, *RF* Rheumatoid factor, *SLE* SYSTEMIC lupus erythematosus, *SLEDAI-2k* SLE disease activity index 2000

### Peripheral blood sampling

Peripheral blood mononuclear cells (PBMCs) were isolated from total blood using Ficoll/Lymphoprep separation (GE Healthcare) and cryopreserved in liquid nitrogen until further use.

### Cell culture and detection of apoptosis

PBMCs were analyzed either directly ex vivo (=unstimulated), or stimulated using 1 µg/ml ODN 2006 (CpG) for B cell stimulation, or CD3 (clone 1XE) and CD28 (clone 15E8) soluble antibodies for T cell stimulation for 6 days. Cell proliferation was measured using a CellTrace violet cell proliferation kit (ThermoFisher Scientific, Waltham, Massachusetts, USA). Proliferation and division index were calculated using the proliferation platform in FlowJo v10 software (BD Biosciences, San Jose, CA, USA). PBMCs were incubated with different Bcl-2 family inhibitors for 24 h. Cell viability was measured by flow cytometry using DiOC6 and TO-PRO-3 viability dyes.

### Reagents

ODN2006 CpG oligonucleotide type B was purchased from InvivoGen (San Diego, CA, USA), venetoclax was purchased from Active Biochem (Bonn, Germany), S-63845 was purchased from Chemgood (Glen Allen, VA, USA), and AZD4320 was provided by Acerta Pharma (Oss, The Netherlands).

### Flow cytometry

PBMCs were stained with the antibodies specific for CD4 (Biolegend, San Diego, CA, USA), CD8 (Biolegend, San Diego, CA, USA), CD19 (BD Biosciences, San Jose, CA, USA), CD27 (BD Biosciences, San Jose, CA, USA), CD38 (BD Biosciences, San Jose, USA), CD45RA (BD Biosciences, San Jose, CA, USA), and IgD (Biolegend, San Diego, CA, USA). Cells were subsequently permeabilized and stained with antibodies targeting Bcl-2 (Biolegend San Diego, CA, USA), Bcl-XL (Cell Signaling Technology, Danvers, Massachusetts, USA), and Mcl-1 (Cell Signaling Technology, Danvers, Massachusetts, USA). PBMCs were measured on an LSR Fortessa (BD Biosciences, San Jose, USA) and analyzed using FlowJo v10.8. Gating strategy of flow cytometry analyses of B and T cell subsets are represented in Supplementary Fig. 1.

### Statistical analysis

Statistical analyses were performed using Graphpad Prism 9 (Graph Pad, San Diego, CA, USA). *T*-tests and two-way analysis of variance (ANOVA) were used to analyze differences between groups. *p < 0.05; **p < 0.01; ***p < 0.001; ****p < 0.0001.

## Results

### Expression of Bcl-2 family members in CD19+ B cell subsets of SLE/RA patients and healthy controls ex vivo and after activation

**Fig. 1:**
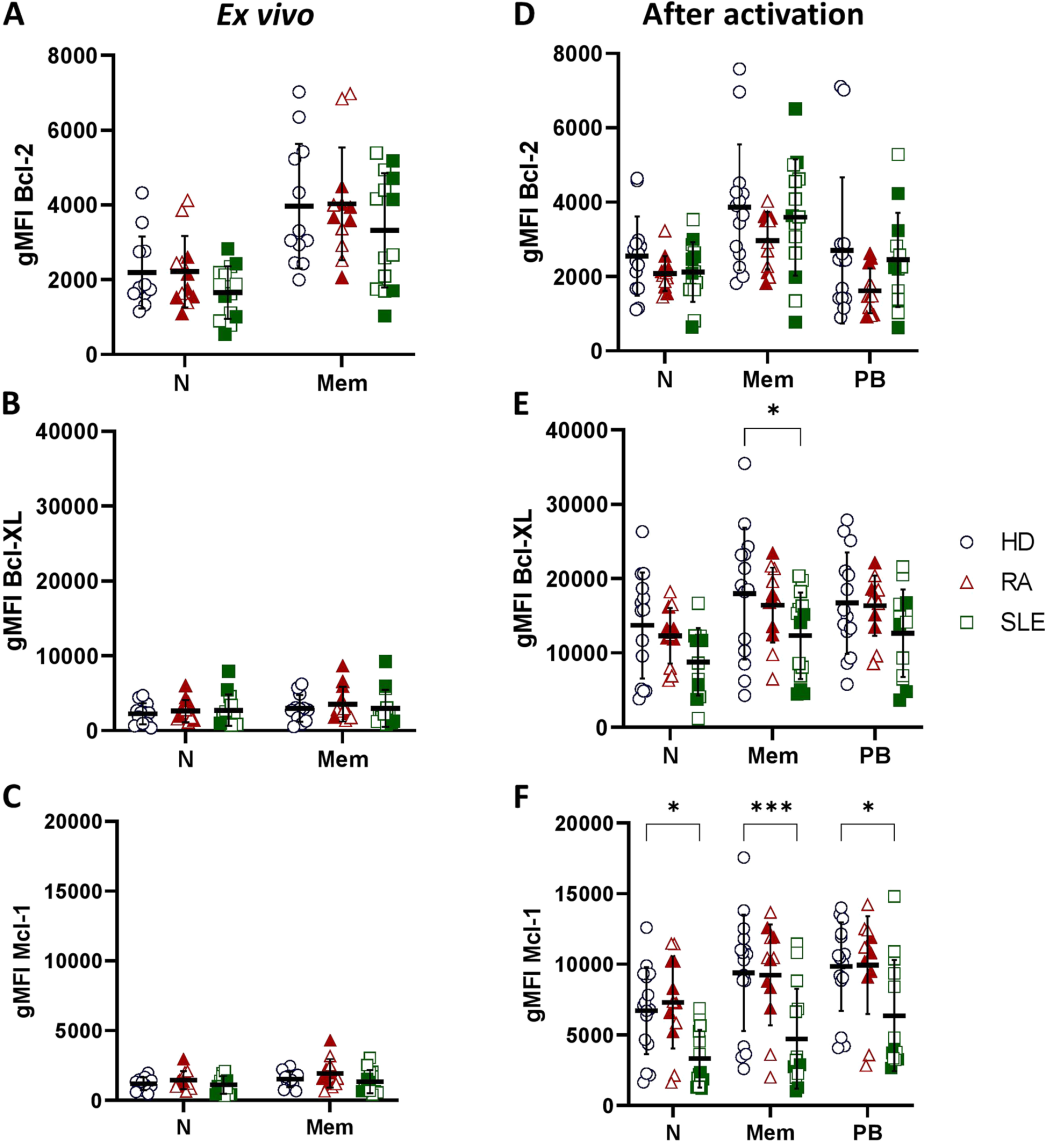
Expression of Bcl-2 family members in CD19+ B cell subsets *ex vivo* and after activation on day 6. Immunological detection of Bcl-2, Bcl-XL, and Mcl-1 expression in CD19+ naïve (N; CD27-/CD38-), memory B cells (Mem; CD27+/CD38-), and plasmablasts (PB; CD27+/CD38+) of healthy controls (HD; *N* = 13) compared to rheumatoid arthritis (RA; *N* = 12), and systemic lupus erythematosus (SLE; *N* = 14) *ex vivo* (**A–C**) and after stimulation with 1 µg/ml ODN2006 (CpG) for 6 days (**D–F**). Filled symbols represent active disease, open symbols remission. Data are expressed as mean ± SD

Detection of Bcl-2 family members by flow cytometry was performed to investigate expression levels in peripheral blood cells of RA/SLE patients compared to healthy controls. *Ex vivo* analyses revealed that there were no statistical differences in *ex vivo* expression of Bcl-2, Bcl-XL, and Mcl-1 between RA/SLE patients and healthy controls in CD19+ B cell subsets (Fig. 1A–C; for primary data and gating strategy, see Supplemental Fig. 1). However, after stimulation with ODN2006 (CpG) for 6 days, there was a fivefold induction of Bcl-XL and Mcl-1 in all B cell subsets of healthy controls and RA/SLE patients (Fig. 1E–F). No significant differences between RA patients and healthy controls were measured. However, there was a significant reduced induction of Bcl-XL in memory (CD27-/CD38+) B cells and an overall significant reduced induction of Mcl-1 in all B cell subsets of SLE patients compared to healthy controls (Fig. 1E–F). These findings were not correlated with disease activity for Bcl-2 and Bcl-XL expression, while active SLE patients (SLEDAI-2k ≥ 6 with a clinical SLEDAI-2k (excluding laboratory results) score ≥ 4) showed reduced Mcl-1 expression compared to SLE patients in remission (Fig. 1F). Taken together, after CpG-induced cell activation B cells from SLE patients show less signs of cellular activation.

### CD19+ B cell proliferation in SLE/RA patients and healthy controls after activation

**Fig. 2:**
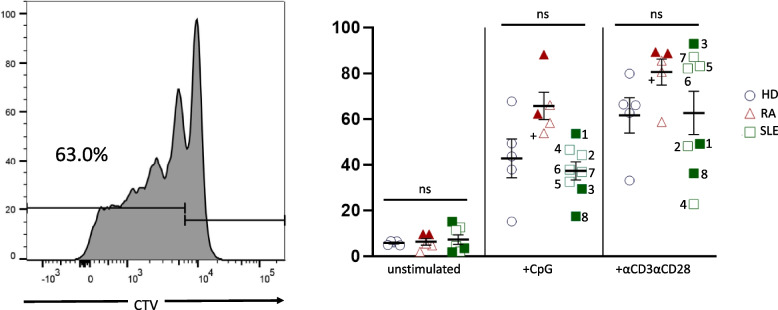
CD19+ B cell proliferation following stimulation for 6 days. Cell proliferation was assessed by a CellTrace violet (CTV) staining in CD19+ B cells of healthy controls (HD) compared to RA/SLE patients after stimulation for 6 days with 1 µg/ml ODN2006 (CpG) or with T cell-dependent stimulation with CD3 (clone 1XE) and CD28 (clone 15E8) soluble antibodies. RA patients were all treated with methotrexate, while one patient also received prednisolone 5 mg/day (+). Two SLE patients were de novo (no mediation; 1+5), while most SLE patients received hydroxychloroquine (HCQ; 2,3,4) or a combination of HCQ with either azathioprine (6) or prednisone 15 mg/day (8), one SLE patient was treated with mycophenolate mofetil (7). Filled symbols represent active disease, open symbols remission. Two-way Anova test was used for statistical analyses; ns = not significant. Data are expressed as mean ± SD.

We next investigated CD19+ B cell proliferation after stimulation for 6 days with CpG or αCD3/αCD28 soluble antibodies to simulate T cell-dependent proliferation. Overall, B cell proliferation was increased after T cell stimulation (Fig. 2), indicating that the release of cytokines by activated T cells can induce B cell proliferation. No differences in cell proliferation were measured between SLE patients and healthy controls, while RA patients showed slightly increased cell proliferation after both stimulations but the calculated proliferation and division index were not significantly different (data not shown). Moreover, these findings did neither correlate with disease activity nor with the use of any medication (Fig. 2).

### Mcl-1 inhibition was the most effective in reducing plasmablast viability after activation

**Figure 3.**
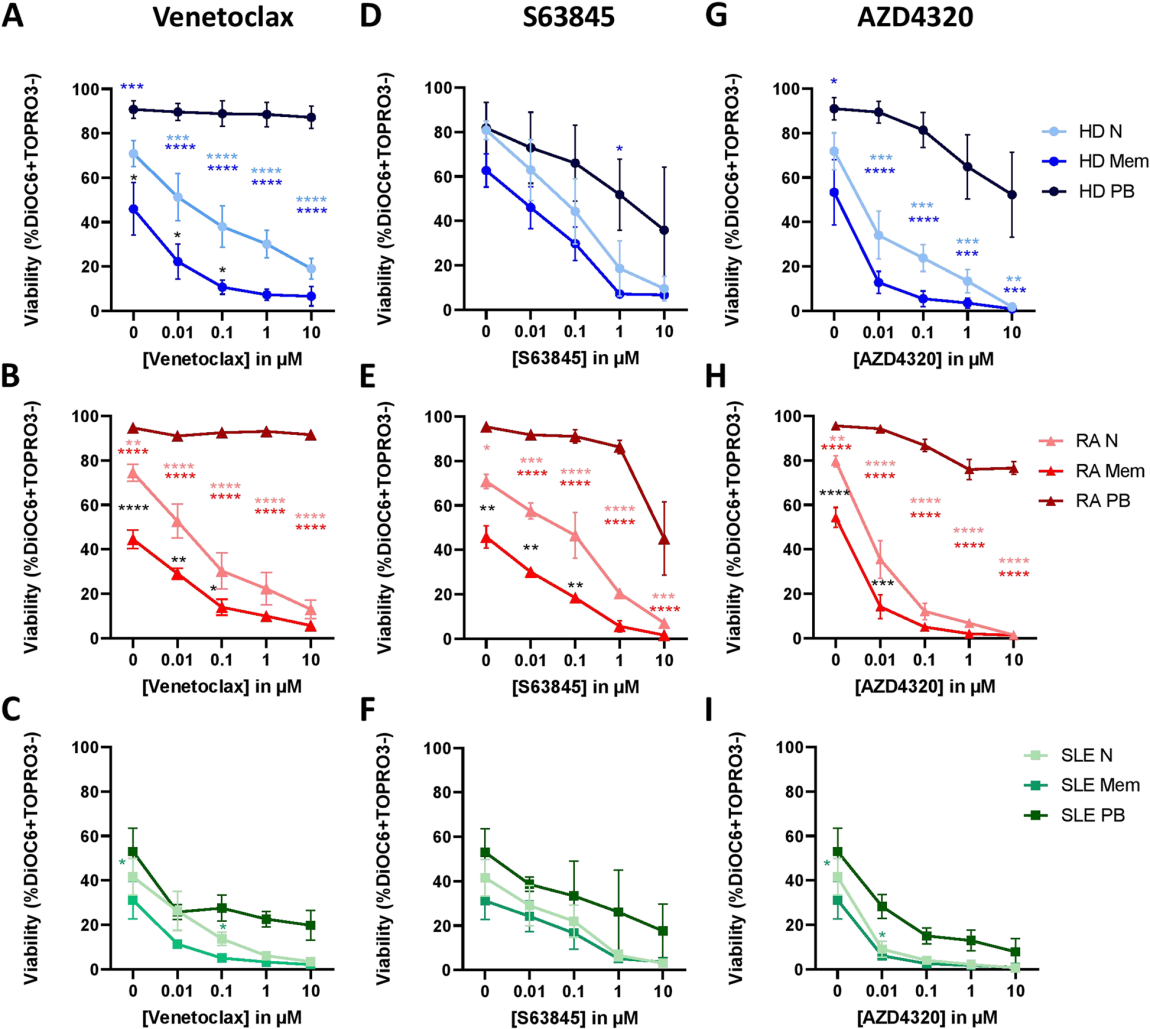
BH3 mimetics revealed that Mcl-1 inhibition was the most effective in reducing plasmablast viability after activation. PBMCs of healthy controls (HD; *N* = 3), rheumatoid arthritis (RA; *N* = 3), and systemic lupus erythematosus (SLE; *N* = 4) were stimulated with 1 µg/ml ODN2006 (CpG) for 6 days followed by *in vitro* treatment with the Bcl-2 inhibitor venetoclax (A–C), Mcl-1 inhibitor S63845 (D–F), or dual Bcl-2/Bcl-XL inhibitor AZD4320 (G–I) for 24 h. Viability data were measured by flow cytometry using DiOC6/TO-PRO-3 staining in CD19+ naïve (N; CD27-/CD38-), memory B cells (Mem; CD27+/CD38-), and plasmablasts (PB; CD27+/CD38+). Two-way Anova test was used for statistical analyses. *p < 0.05; **p < 0.01; ***p < 0.001. Data are expressed as mean ± SEM

Long-lived memory-effector cells or plasma cells are assumed to contribute to disease manifestations in SLE and RA. Since Bcl-2 family inhibitors (BH3 mimetics) might be novel therapeutics for the treatment of both autoimmune diseases, we studied their effect on different B cell subsets. After *in vitro* stimulation with CpG for 6 days, plasmablasts were more viable after treatment with Bcl-2 inhibitors for 24 h compared to naïve or memory B cells of healthy controls and autoimmune diseases (Fig. 3). Single Bcl-2 inhibition using venetoclax showed no effect on plasmablast viability at all, while dual Bcl-2/Bcl-XL inhibition using AZD4320 reduced viability of all B cell subsets even at lower concentrations (Fig 3G-I). Mcl-1 inhibition using S63845 was most effective in reducing plasmablast viability compared to the other Bcl-2 inhibitors. However, for both compounds AZD4320 and S63845, higher concentrations were needed to induce the same effects in PB cells from patients with autoimmune diseases as those observed with healthy control cells. Along various experiments, the cell viability of SLE patients was reduced after 6 days of CpG stimulation compared to healthy controls or RA patients. Therefore, the results of SLE patients cannot be easily compared to healthy controls or RA patients upon addition of BH3 inhibitors at day 6. In order to investigate whether these differences in cell survival between patient groups were due to variation in freezing/thawing methods, we tested various patient samples to rule out this possibility. Moreover, primary cells of analyzed patient samples were still viable after 24 h which makes it less likely to explain these observations by methodological issues (data not shown). Due to the lack of patient material, we focused on BCR-independent stimulation and could not study other B cell proliferation-inducing stimuli simultaneously, such as BCR stimulation via α-IgM/α-CD40/IL-21. We also measured apoptosis in unstimulated samples after addition of BH3 mimetics for 24 h and we found similar results as seen in Fig. 3 for naïve and memory B cells. However, there were not enough plasmablasts to analyze apoptosis in this subset (data not shown).

Taken together, these data imply that, of these three Bcl-2 family members, Mcl-1 expression is the most important in plasmablast survival, and that this dependence on Mcl-1 is further increased in case of RA plasmablasts.

### Expression of Bcl-2 family members in CD4+/CD8+ T cell subsets of SLE and RA patients ex vivo and after activation

**Fig. 4:**
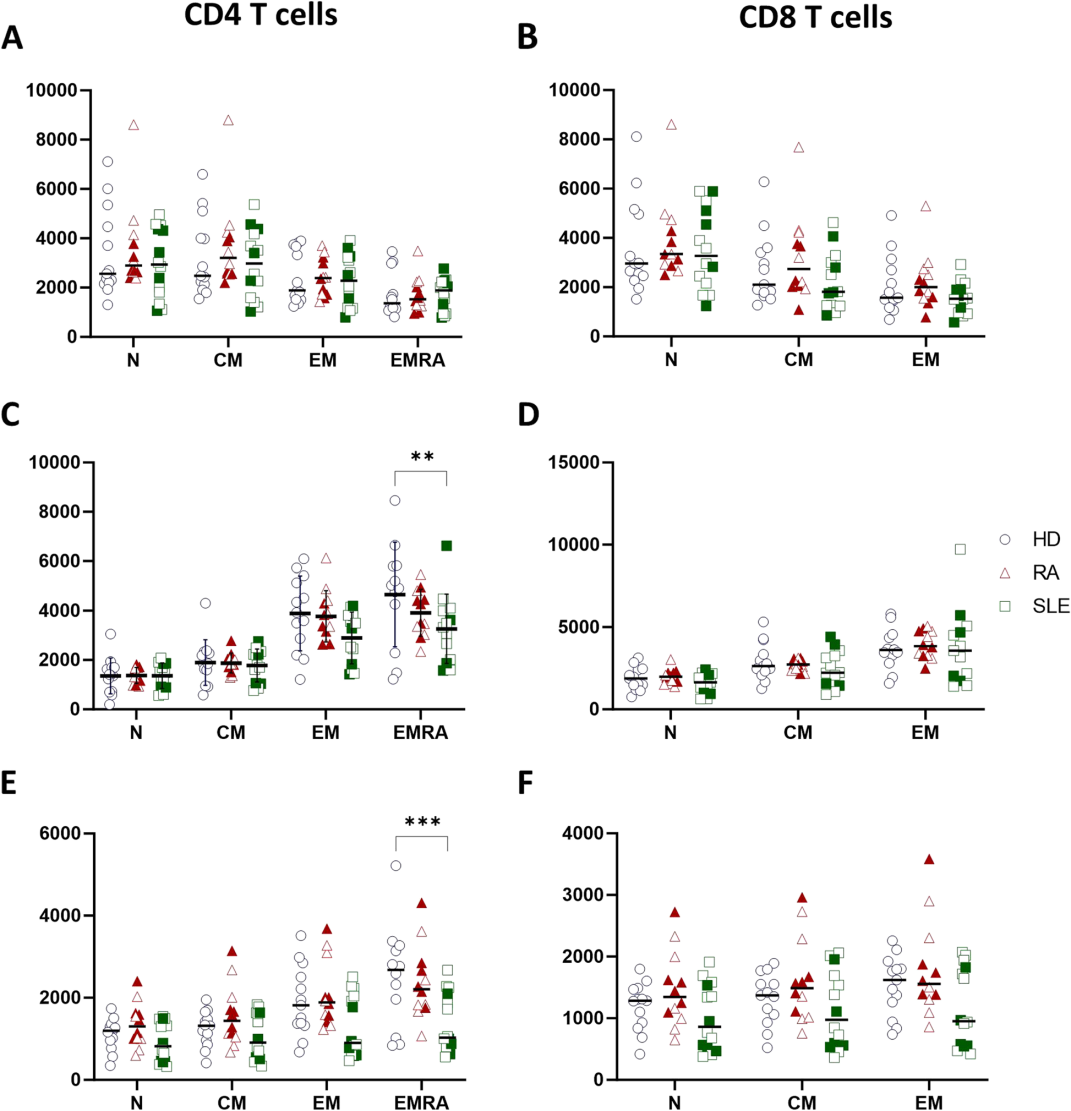
Expression of Bcl-2 family members in T cell subsets *ex vivo*. Flow cytometric analysis of Bcl-2 (**A–B**), Bcl-XL (**C–D**), and Mcl-1 expression (**E–F**) in CD4+ and CD8+ naïve (N; CD27+/CD45RA+), central memory (CM; CD27+/CD45RA-), effector memory (EM; CD27-/CD45RA-), and effector memory cells re-expressing CD45RA (EMRA; CD27-/CD45RA+) T cell subsets of healthy controls (HD; *N* = 13) compared to rheumatoid arthritis (RA; *N* = 12) and systemic lupus erythematosus (SLE; *N* = 14) *ex vivo*. Filled symbols represent active disease, open symbols remission. Two-way Anova test was used for statistical analyses. *p < 0.05; **p < 0.01; ***p < 0.001. Data are expressed as mean ± SD

**Fig. 5:**
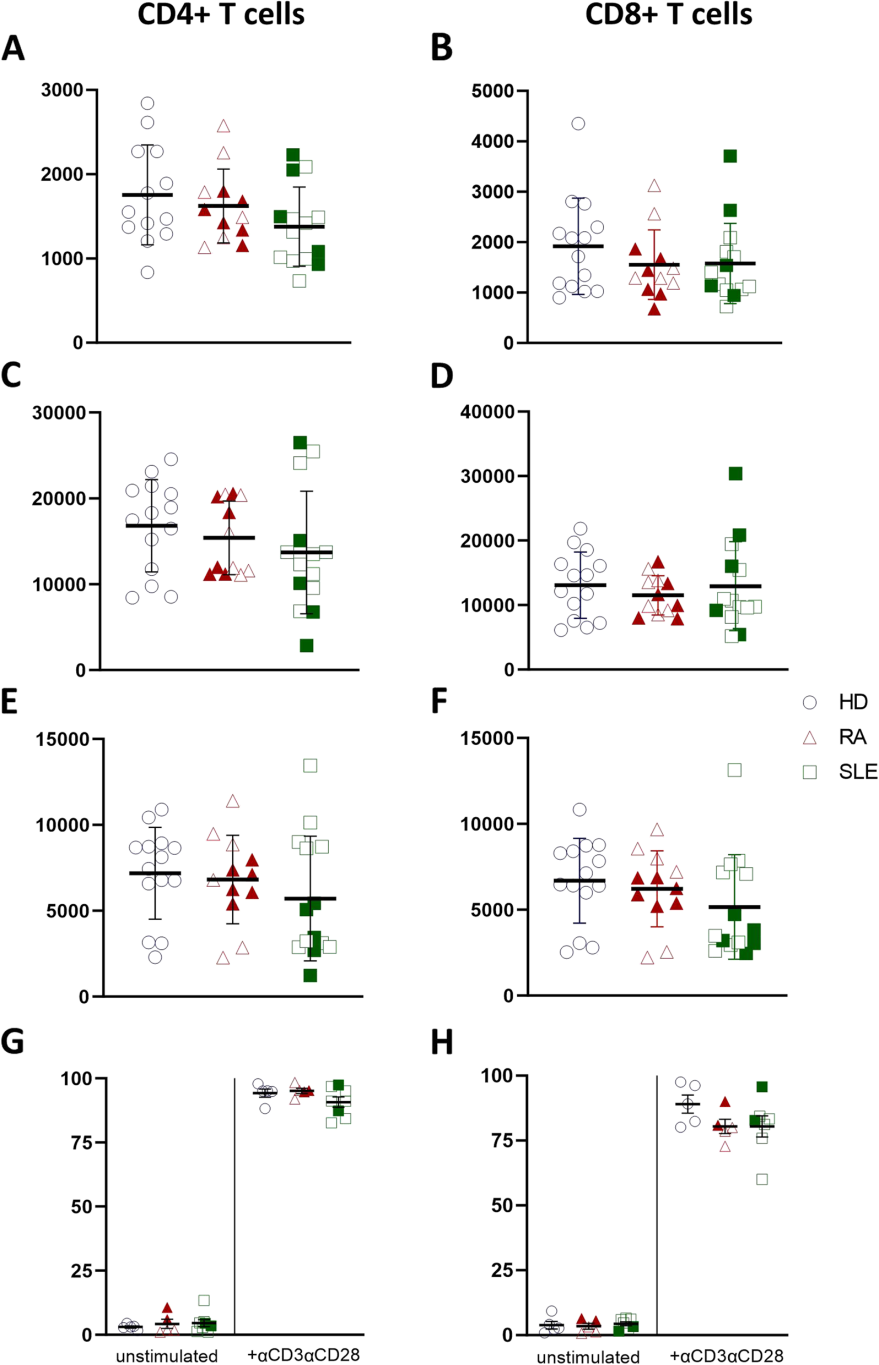
Expression of Bcl-2 family members in T cells after activation on day 6. Flow cytometric analysis of Bcl-2 (**A–B**), Bcl-XL (**C–D**), Mcl-1 expression (**E–F**), and cell proliferation (**G–H**) in total CD4+ or CD8+ T cell populations of healthy controls (HD; *N* = 13) compared to rheumatoid arthritis (RA; *N* = 12) and systemic lupus erythematosus (SLE; *N* = 14) after stimulation with CD3 (clone 1XE) and CD28 (clone 15E8) soluble antibodies for 6 days. Filled symbols represent active disease, open symbols remission. Data are expressed as mean ± SD

We also investigated expression levels of Bcl-2 family members in T cells. Immunological detection of Bcl-2 family proteins in CD4+/CD8+ T cell subsets revealed that Bcl-XL and Mcl-1 were significantly reduced in effector memory CD4+CD27^neg^CD45RA+ (EMRA) (for primary gating strategy, see Supplemental Fig. 1B) T cells of SLE patients compared to healthy controls *ex vivo* (Fig. 4C, E). There were no differences in expression levels of Bcl-2 family members in RA patients detected compared to healthy controls (Fig. 4). After stimulation with a combination of αCD3/CD28 soluble antibodies for 6 days, there was an overall induction of Bcl-XL and Mcl-1 in the total T cell populations (Figs. 4 and 5) but no differences in expression levels of Bcl-2 family members were measured in SLE/RA patient samples and healthy controls (Fig. 5). Further subset analysis was not reliable after activation (Supplemental Fig. 1C). Similar to activated B cells, SLE patients with active disease (SLEDAI-2k ≥ 6 with a clinical SLEDAI-2k (excluding laboratory results) score ≥ 4) showed decreased Mcl-1 expression in both CD4+/CD8+ T cell populations after activation (Fig. 5E–F). We also noted some variation in Mcl-1 expression among patients. Concerning proliferation of T cells after activation, no differences in cell proliferation of RA or SLE patients compared to healthy controls were detected in CD4+ T cells, while CD8+ T cell proliferation was slightly reduced in both autoimmune diseases when compared to controls (Fig. 5G–H).

### Mcl-1 inhibition was the most effective in reducing both CD4+ and CD8+ T cells viability after activation in healthy controls

**Fig. 6:**
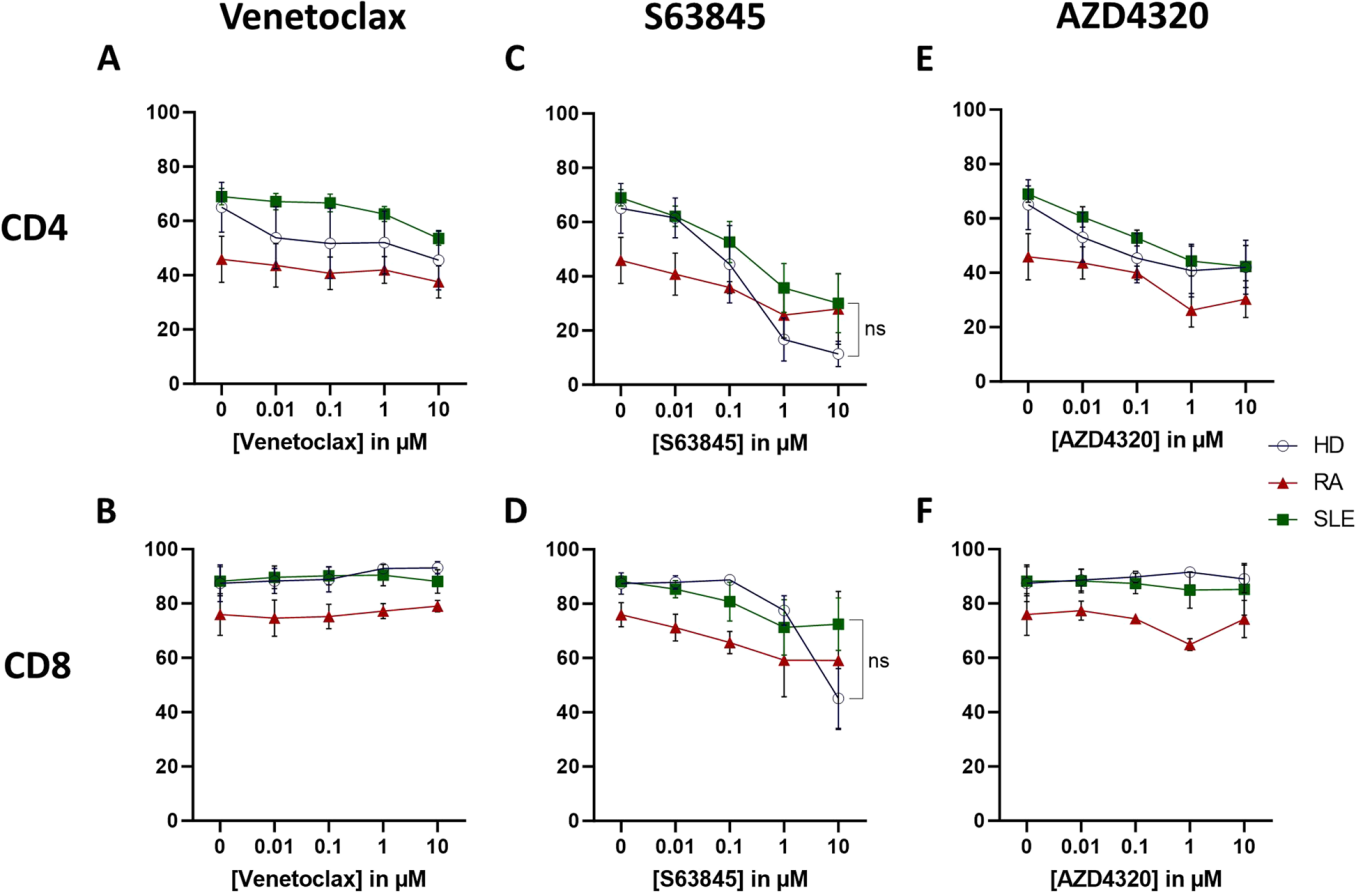
Mcl-1 inhibition was the most effective in reducing viability of both CD4+ and CD8+ T cells after activation in healthy controls. PBMCs of healthy controls (HD; *N* = 3), rheumatoid arthritis (RA; *N* = 3), and systemic lupus erythematosus (SLE; *N* = 7) were stimulated with CD3 (clone 1XE) and CD28 (clone 15E8) soluble antibodies for 6 days followed by in vitro treatment with the Bcl-2 inhibitor venetoclax (**A–B**), Mcl-1 inhibitor S63845 (**C–D**) or dual Bcl-2/Bcl-XL inhibitor AZD4320 (**E–F**) for 24 h. Viability data were measured by flow cytometry using DiOC6/TO-PRO-3 staining in total CD4+ or CD8+ T cell populations. Two-way Anova test was used for statistical analyses; ns not statistically significant. Data are expressed as mean ± SEM

Finally, we investigated the effects of Bcl-2 family inhibitors in activated CD4+ and CD8+ T cells. While we previously noticed decreased B cell viability in SLE patients, we found that in RA patients upon activation viability of CD4+ T cells was reduced instead, compared with cultures in SLE patients or healthy controls (Fig. 6). Remarkably, none of the tested Bcl-2 family inhibitors reduced CD4+/CD8+ T cell viability in RA patients, whereas high concentrations of Mcl-1 inhibition led to an approximately 30% reduction in CD4+ T cell viability in SLE. However, the effect of Mcl-1 inhibition in inducing cell death was most prominent in both CD4+/CD8+ T cells of healthy controls compared to autoimmune diseases (Fig. 6C, D). Nevertheless, these differences were not statistically significant.

## Discussion

To date, limited data are available on the expression pattern of Bcl-2 family members in patients with autoimmune diseases like SLE and RA. In this in vitro study, we show evidence for a differential expression pattern of Bcl-2 family members in B and T cell subsets of patients with SLE and RA compared to healthy controls, consisting of aberrant expression in the B cell compartment upon activation in SLE. Previous studies showed that overexpression of anti-apoptotic Bcl-2 in transgenic mice led to an increase in lifespan of plasma cells [7], contributing to the production of auto-antibodies which are known to play a key role in the pathogenesis of SLE [22]. However, in this study, we demonstrated that there were no significant differences in the expression of Bcl-2 family members between SLE/RA patient samples and healthy controls, unless activated. Moreover, we found that both Bcl-XL and Mcl-1 protein levels showed less induction in SLE compared to healthy controls. Especially in active SLE patients (SLEDAI-2k ≥ 6 with a clinical SLEDAI-2k (excluding laboratory results) score ≥ 4), Mcl-1 expression was decreased after stimulation with CpG for 6 days, which supports previous studies showing that active SLE is associated with reduced cytokine production, proliferation and activation upon stimulation with a TLR9 agonist compared to healthy controls [23]. For patients with RA, we did not find any statistical differences in Bcl-2, Bcl-XL, and Mcl-1 expression regarding the different B cell subsets at basal levels and upon activation. A reasonable explanation for this difference is that we examined the expression of the Bcl-2 family members in lymphocytes recruited from peripheral blood, but the major location of the inflammation is localized in the joints. As in the majority of the included RA patients (92%), we did not notice any extra-articular manifestations (Table 2). A few attempts have been made to study Bcl-2 expression in lymphocytes from synovial fluid or synovial tissue [24–26], but unfortunately, the results were contradictory. Another possibility for our findings is that we studied the expression level on the total population of B lymphocytes instead of focusing on the minute fraction of autoreactive, anti-CCP expressing B cells.

Similar to B cells, we found decreased Bcl-XL and Mcl-1 expression in CD4+ terminally differentiated effector memory T cells in SLE patient samples compared to healthy controls ex vivo. Upon 6 days of activation, there were no significant differences in expression levels of Bcl-2 family members in the overall population of total T cells. We did not find signs of general overexpression of Bcl-2 family members in autoimmune diseases compared to healthy controls, but rather reduced Bcl-XL and Mcl-1 protein levels in SLE cell subsets.

Our study has several limitations. It included a relatively small number of patients and our study is in vitro by nature. However, the data on this topic is still very limited and findings from this study can provide context and guidance for future research. Furthermore, our study is performed on B and T lymphocytes recruited from the peripheral blood. It would be of great interest to select only the autoantigen-specific lymphocytes within such cell preparations, although this will be experimentally challenging as these autoreactive cells may represent a small subset of the total B and T cell populations demanding large blood volumes for analysis. Additionally, its role in other immune cells (and recruited from other locations, e.g., tissue/synovium) needs to be further elucidated as there is scientific evidence that increased expression of MCL-1 in synovial fibroblasts and synovial macrophages or an increased expression of BCL-XL in osteoclasts contributed to disease progression in RA [27–29]. Unfortunately, we did not distinguish different T cell subsets due to unreliable surface marker expression after stimulation (see Supplementary Fig. 1).

In this study, we only investigated three members of the Bcl-2 family. As the ratio of the anti- and pro-apoptotic members sets the threshold for susceptibility of apoptosis, a study on the expression levels of the complete set of Bcl-2 family proteins would be interesting as a next step. Furthermore, it cannot be ruled out that there was some unintended bias due to the effect of medication on Bcl-2 family member expression levels.

Gaining more insight in the mechanisms of apoptosis in auto-immune diseases like SLE and RA is highly relevant since a wide array of compounds, termed Bcl-2 homology 3 (BH3) mimetics, have been developed to treat hemato-oncology patients. There is increasing evidence that these BH3 mimetics may also serve as novel therapeutics in auto-immune disease. One of the first examples of a well-studied BH3 mimetic is ABT-199 (Venetoclax), a small molecule that selectively inhibits Bcl-2. Several studies have been published on the safety and pharmacodynamics/kinetics of ABT-199 in SLE patients [30, 31]. Furthermore, it was shown that there is an exposure-dependent reduction of B lymphocyte and total lymphocyte counts in women with SLE treated with ABT-199 [32]. Another Bcl-2 family protein inhibitor is ABT-737, which targets both Bcl-2 and Bcl-XL, thereby reducing disease activity in a CIA mouse model, and a significant protection of severe glomerulonephritis with prolonged survival in the animal model of SLE [33]. However, treatment with ABT-737 was associated with dose-limiting thrombocytopenia in chronic lymphocytic leukemia patients [34].

In this in vitro study, we tested in SLE and RA patient samples the effect on T and B cell activation and proliferation of a novel dual Bcl-2/Bcl-XL inhibitor, AZD4320, which induces tumor regression in hematologic cancer models without dose-limiting thrombocytopenia [18], and compared this compound to venetoclax and a Mcl-1 inhibitor, S63845. While venetoclax was unable to reduce plasmablast viability, dual Bcl-2/Bcl-XL inhibition via AZD4320 or Mcl-1 inhibition via S63845 led to reduced plasmablast viability. Of note, plasmablasts of healthy controls were more sensitive to the BH3 mimetics compared to patient cells, implying that S63845 will not be more effective in autoimmune lymphocytes versus normal cells. Overall, control cell viability of SLE patients was reduced after stimulation with CpG for 6 days followed by overnight treatment with BH3 mimetic drug. As mentioned above, a possible explanation could be that B cells of active SLE showed reduced proliferation and activation upon stimulation with a TLR9 agonist. It was previously shown that SLE patients display increased expression levels of TLR9 [35], which might lead to an overcompensation/overstimulation of lymphocytes in SLE patient samples when stimulating cells for 6 days with CpG. In contrast, we observed that T cell viability of SLE patients after stimulation with αCD3/αCD28 soluble antibodies for 6 days was comparable to healthy controls. Similar to our B cell data, we found that only Mcl-1 inhibition was able to reduce T cell viability in healthy controls but not in RA and to a lesser extent in SLE patients.

## Conclusions

In conclusion, our study provides an overview of expression patterns of Bcl-2 family members in patients with SLE and RA compared to healthy controls in vitro. Since we did not find any overexpression of Bcl-2 family members in peripheral blood of RA/SLE patient samples and our BH3 mimetic results showed more effects in healthy controls, we have not yet sufficient data to support the use of any of the tested BH3 mimetic drugs in the treatment of autoimmunity. Furthermore, some contradictory case reports have been published about the development of autoimmune hemolytic anemia (AIHA) during venetoclax treatment in chronic lymphocytic leukemia (CLL) [36, 37], while other cases reported that venetoclax was beneficial in treating AIHA or refractory immune thrombocytopenia (ITP) in CLL [38, 39]. Nevertheless, some intriguing differences between healthy controls and patients were noted that deserve further study. Our studies included only three Bcl-2 family members. Examination of the complete set of Bcl-2 family members on phenotypically selected cellular subsets in peripheral blood versus tissue (e.g., synovial tissue) may be required in order to make further statements on therapy responsiveness in selected patients.

### Supplementary Information

**Additional file 1:** **Supplementary Figure 1.** Gating strategy of flow cytometric analyses of B and T cell subsets. (A) Gating strategy of flow cytometric analyses for B cell subsets *ex vivo *and after stimulation with 1µg/ml ODN2006 (CpG) for six days. (B) Gating strategy of flow cytometric analyses for CD4 and CD8 T cell subsets *ex vivo. *(C) After stimulation with αCD3/CD28 soluble antibodies, total CD4/CD8 populations have been analysed, since T cell subset analyses were not reliable after activation.

## Data Availability

Patient information and flow cytometry data are available upon reasonable request from the corresponding author.

## Abbreviations

ACR: American College of Rheumatology
ANA: Anti-nuclear antibodies
anti-CCP: Anti-cyclic citrullinated peptide
Bcl-2: B cell lymphoma-2
Bcl-XL: B cell lymphoma Extra-Large
BH3: Bcl-2 Homology 3
CIA: Collagen induced arthritis
DAS-28: Disease Activity Score-28
cDMARD: Conventional disease-modifying antirheumatic drug
Mcl-1: Myeloid cell leukemia-1
MFI: Mean fluorescence intensity
PBMC: Peripheral blood mononuclear cell
RA: Rheumatoid arthritis
RF: Rheumatoid factor
SLE: Systemic lupus erythematosus
SLEDAI-2k: SLE Disease Activity Index 2000
SLICC: Systemic Lupus International Collaborating Clinics
