# Supplementary material for: Differential expression pattern of Bcl-2 family members in B and T cells in systemic lupus erythematosus and rheumatoid arthritis
Source: Arthritis Res Ther. 2023 Nov 22;25:225. doi: 10.1186/s13075-023-03203-7 (PMC10664305; doi:10.1186/s13075-023-03203-7)
Supplement: Supplementary file 1 — Additional file 1: Supplementary Figure 1. Gating strategy of flow cytometric analyses of B and T cell subsets. (A) Gating strategy of flow cytometric analyses for B cell subsets ex vivo and after stimulation with 1µg/ml ODN2006 (CpG) for six days. (B) Gating strategy of flow cytometric analyses for CD4 and CD8 T cell subsets ex vivo. (C) After stimulation with αCD3/CD28 soluble antibodies, total CD4/CD8 populations have been analysed, since T cell subset analyses were not reliable after activation. [file 13075_2023_3203_MOESM1_ESM.docx]

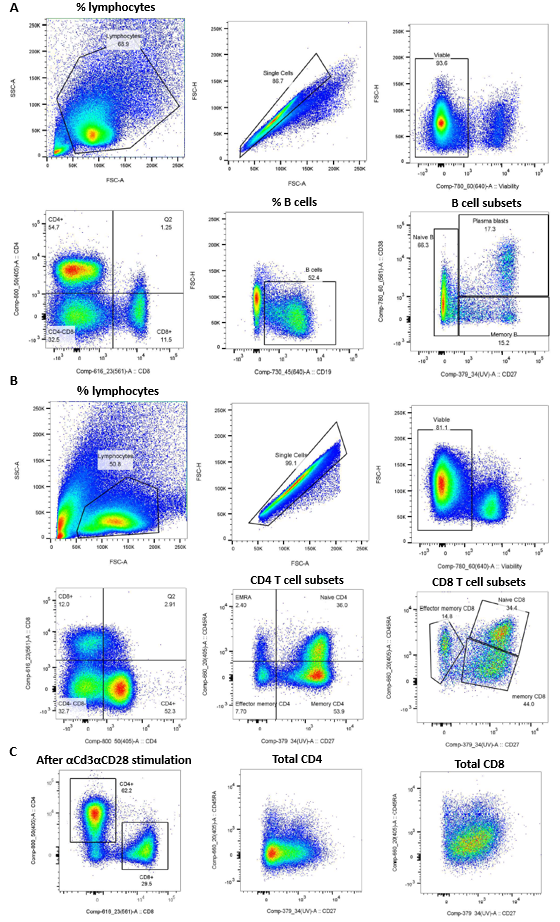
**SUPPLEMENTARY MATERIAL**

**Supplementary Figure 1. Gating strategy of flow cytometric analyses of B and T cell subsets.**
(A) Gating strategy of flow cytometric analyses for B cell subsets *ex vivo* and after stimulation with 1µg/ml ODN2006 (CpG) for six days. (B) Gating strategy of flow cytometric analyses for CD4 and CD8 T cell subsets *ex vivo.* (C) After stimulation with αCD3/CD28 soluble antibodies, total CD4/CD8 populations have been analysed, since T cell subset analyses were not reliable after activation.
